# Effects of Confined Microenvironments with Protein Coating, Nanotopography, and TGF-β Inhibitor on Nasopharyngeal Carcinoma Cell Migration through Channels

**DOI:** 10.3390/jfb15090263

**Published:** 2024-09-11

**Authors:** Xiao Hong, Yuanhao Xu, Stella W. Pang

**Affiliations:** 1 Department of Electrical Engineering, City University of Hong Kong, Hong Kong, China; xiaohong3-c@my.cityu.edu.hk (X.H.); yuanhaoxu2@cityu.edu.hk (Y.X.); 2 Centre for Biosystems, Neuroscience, and Nanotechnology, City University of Hong Kong, Hong Kong, China

**Keywords:** nasopharyngeal carcinoma (NPC), microwells with confined channels, nanohole topography

## Abstract

Distant metastasis is the primary cause of unsuccessful treatment in nasopharyngeal carcinoma (NPC), suggesting the crucial need to comprehend this process. A tumor related to NPC does not have flat surfaces, but consists of confined microenvironments, proteins, and surface topography. To mimic the complex microenvironment, three-dimensional platforms with microwells and connecting channels were designed and developed with a fibronectin (FN) coating or nanohole topography. The potential of the transforming growth factor-β (TGF-β) inhibitor (galunisertib) for treating NPC was also investigated using the proposed platform. Our results demonstrated an increased traversing probability of NPC43 cells through channels with an FN coating, which correlated with enhanced cell motility and dispersion. Conversely, the presence of nanohole topography patterned on the platform bottom and the TGF-β inhibitor led to a reduced cell traversing probability and decreased cell motility, likely due to the decrease in the F-actin concentration in NPC43 cells. This study highlights the significant impact of confinement levels, surface proteins, nanotopography, and the TGF-β inhibitor on the metastatic probability of cancer cells, providing valuable insights for the development of novel treatment therapies for NPC. The developed platforms proved to be useful tools for evaluating the metastatic potential of cells and are applicable for drug screening.

## 1. Introduction

Nasopharyngeal carcinoma (NPC) is a subtype of head and neck cancers, distinguished by its high potential for invasiveness and metastasis [1,2]. By far, distant metastasis remains the foremost cause of mortality among NPC patients [3,4], underscoring the pressing need for a better understanding of NPC metastasis.

Cancer metastasis is a multiple-step process that entails tumor growth and expansion at the primary site, invasion into surrounding blood vessels, migration and circulation to distant organs, extravasation from the vessels, and ultimately survival and development at secondary sites [5,6]. Throughout these steps, cancer cells encounter intricate microenvironmental changes, both biophysically and biochemically. In natural tissues, the extracellular matrix (ECM) forms a three-dimensional (3D) network of macromolecular mixtures that occupy the extracellular space between cells, creating a confined microenvironment around cells, while also providing structural support for cell adhesion and migration [7,8]. The degree of confinement created by the ECM varies between different tissues, resulting in different ECM interactions with cells during the metastasis process as they transition from the primary site to the secondary site. Previous studies have demonstrated that the confined environment has a profound effect on cancer cell adhesion, motility, and invasiveness [9,10,11], indicating its critical role in the cancer metastasis process. In addition, the protein composition and topography of the ECM could also regulate cell behavior. For instance, fibronectin (FN), one of the main components of the basement membrane in the ECM [12], has been shown to promote tumor progression by enhancing cell motility, proliferation, and the epithelial–mesenchymal transition [13,14,15]. Moreover, the topography of the ECM at both micrometer and nanometer levels has been shown to have significant effects on the regulation of cell functions. These effects encompass crucial aspects such as the cell metastasis potential, cell polarity, and immune response [16,17,18].

Taken together, these findings highlight the significance of ECM confinement, surface proteins, and topography in the progression of cancer. Although the use of the traditional 2D culture system has contributed valuable insights into the molecular mechanism underlying cancer metastasis, it is significantly different from complex 3D ECM structures, leading to striking differences between 2D culture models and clinical trials during drug testing [19,20]. Currently, Transwell and Matrigel assays are widely employed to investigate cancer cell migration and invasion [21,22]. Despite their usefulness in elucidating cancer cell behaviors in a 3D microenvironment, these platforms still have certain limitations. One notable limitation is the absence of real-time monitoring capabilities, which restricts the observation and analysis of cell behavior during translocation. Moreover, these platforms lack precise and flexible control over the dimensions and topographies of microenvironments, hindering their ability to accurately replicate the complexity of 3D tumors. To overcome these limitations, the development of novel cell culture platforms that can closely represent 3D ECM structures is imperative, which could provide a more comprehensive understanding of the intricate factors governing cancer metastasis and drive advancements in this field. With the rapid development of micrometer and nanometer fabrication technologies, 3D engineered platforms with precisely controlled shapes and dimensions have shown promising potential in regard to addressing the aforementioned challenges. These platforms were typically made of transparent polydimethylsiloxane (PDMS), which allowed the real-time imaging of live cells and yielded significant advances in the study of cancer cell migration [23,24,25,26] and drug screening [27,28].

In this work, biomimetic engineered platforms consisting of microwells and connecting channels, along with various surface modifications, were designed and fabricated to investigate the metastatic behaviors of NPC cells. The integration of biochemical modification and novel nanotopography patterning into the platforms provides more realistic models to explore cell responses to multiple aspects of the microenvironment’s influence. The microwells and channels had varying dimensions. The microwells were 50 × 50 and 100 × 100 μm^2^ in size to mimic an early-stage NPC tumor, typically ranging from 50 to 250 μm [29,30]. The channels resembled the ECM and blood vessels with widths of 10, 20, and 30 μm, reflecting the ECM confinement and the small sizes of the arteries, veins, and capillary vessels ranging from 5 to 30 μm [31]. To trap NPC cells, the depth of the microwells and connecting channels was set at 50 μm, which provided effective confinement within the microwells and channels. This configuration allowed the NPC cells to migrate through a series of microenvironments, transitioning from a loosely confined environment in a microwell to a more tightly confined channel and then to another loosely confined environment in a microwell, thereby simulating a simplified metastasis process. The platform was further enhanced by being coated with an ECM protein or patterned with nanohole topography to provide the ECM with a different chemical and physical complexity. Additionally, this study demonstrated the application of the platform for anti-cancer drug screening by treating NPC cells with the transforming growth factor-β (TGF-β) inhibitor. With various modifications of the platforms, the NPC cells exhibited different responses to the microenvironments, including the traversing probability through narrow channels, cell motility, and cell morphology. The findings presented in this work provide valuable insights into the interactions between cells and their microenvironment, significantly advancing our knowledge about cancer cell migration in confined microenvironments, with different biochemical and biophysical modifications. The application of this novel, 3D biomimetic platform for anti-cancer drug screening demonstrates its potential in evaluating therapeutic strategies. 

## 2. Materials and Methods

### 2.1. Microfabrication of Microwells and Connecting Channels with Nanoholes on the Bottom of PDMS Platforms

**Figure 1:**
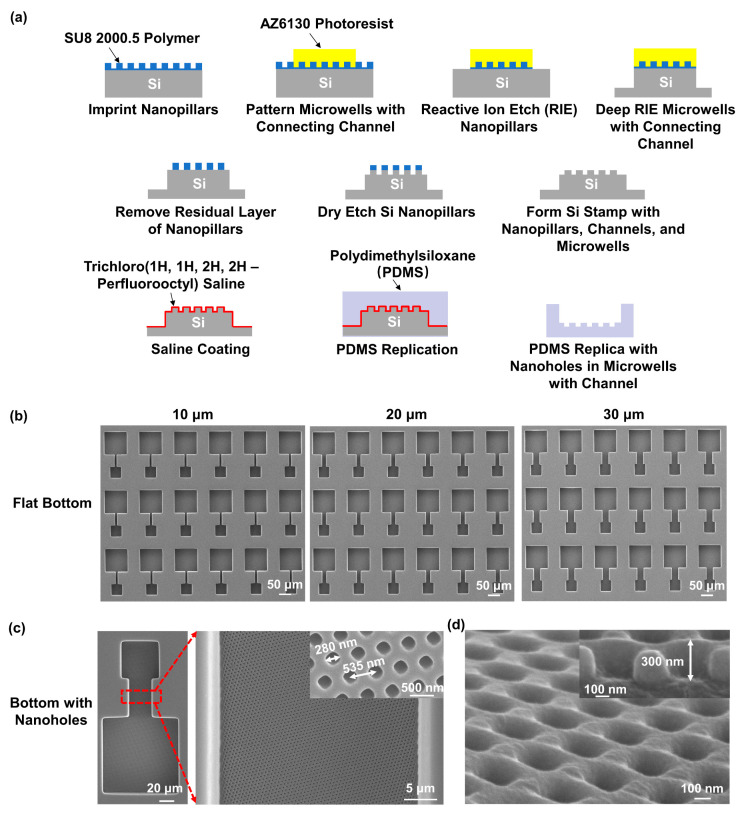
Fabrication of microwells with connecting channels and nanoholes in polydimethylsiloxane (PDMS). (**a**) Schematics of fabrication technology used to replicate PDMS platform with microwells, connecting channels, and nanoholes from Si stamp. (**b**) Micrographs of microwell and connecting channel arrays with flat bottom. Channels had widths of 10, 20, and 30 μm, and were 50 μm deep. (**c**) Micrographs of microwells and 30 μm wide connecting channel with nanohole bottom. Nanoholes in microwells and channel had width of 280 nm and period of 535 nm. (**d**) Nanoholes with a height of 300 nm.

Figure 1a illustrates the fabrication technology used to create a pattern of nanoholes on the bottom of the microwells and connecting channel array on the PDMS (SYLGARDTM 184, Dow, Midland, MI, USA) platforms. Firstly, a silicon (Si) wafer was cleaned in an ultrasonic bath using acetone, isopropanol, and deionized (DI) water, for 10 min each. The wafer was then dried and treated with an O_2_ plasma to increase its hydrophilicity. Next, SU8 2000.5 photoresist (MicroChem, Westborough, MA, USA) was spin coated onto the wafer, at 3000 revolutions per min, for 1 min, and baked at 65 °C and 95 °C, for 2 min each. The SU8 nanopillars, with a diameter of 280 nm and period of 535 nm, were nanoimprinted using an intermediate polymer stamp (IPS), fabricated by being imprinted from a nickel stamp, with the conditions of 40 bar and 150 °C for 5 min. The IPS was coated with 97% trichloro (1H,1H,2H,2H-perfluorooctyl) silane (FOTS, J&K Scientific, Beijing, China) to facilitate demolding from the SU8 nanopillars. The microwells with connecting channels were then set out in a pattern on top of the SU8 nanopillars, using photolithography with AZ6130 photoresist, followed by reactive ion etching (RIE) to remove the nanopillars not covered by the AZ6130 photoresist. The RIE conditions were 2/20 sccm SF_6_/O_2_, 10 mTorr, and 100 W radio frequency (rf) power for 5 min. Deep RIE (DRIE) was then used to etch the Si wafer, to achieve a depth of 50 μm for the microwells and connecting channels. After removing the AZ6130 photoresist, the residual layer around the SU8 nanopillars was removed by RIE, with the conditions of 2/20 sccm SF_6_/O_2_, 10 mTorr, and 100 W rf power for 1 min. Another DRIE was conducted to etch the Si nanopillars. After removing the SU8 photoresist, the Si stamp with nanopillars, channels, and microwells was achieved.

The PDMS replicas, with nanoholes in the microwells and connecting channel array, were then peeled off from the Si stamp. To prevent the PDMS from sticking to the Si stamp, the stamp was coated with FOTS at 80 °C for 20 min. The PDMS prepolymer mixed with a silicone elastomer base and curing agent at a weight ratio of 10:1 was poured onto the Si stamp. After degassing in a vacuum chamber for 1 h, the PDMS was cured at 25 °C for 12 h and 80 °C for 4 h.

The fabrication technology for the PDMS replica, containing the microwells and connecting channel array with a flat bottom, was similar to that described above. Briefly, the microwells and connecting channels were patterned on a Si wafer with AZ6130 photoresist using photolithography. The Si wafer was then etched in the DRIE system to achieve 50 μm deep microwells and channels. After removing the residual AZ6130 photoresist, a Si stamp consisting of microwells and connecting channels with a flat bottom was achieved. The PDMS replica was peeled off from the Si stamp using similar technologies as described above.

The fabricated PDMS platform, containing microwells with connecting channels, is shown in Supplementary Figure S1. The detailed structures of the microwells and connecting channels with flat bottom surfaces are shown in Figure 1b. The platforms comprised two 50 μm deep microwells that were 50 × 50 and 100 × 100 μm^2^ in size and that were connected by a 50 μm long channel. The channels had widths of 10, 20, and 30 μm. Figure 1c shows micrographs of the microwells and 30 μm wide connecting channel with the nanoholes at the bottom, along with an enlarged image. The nanoholes on the bottom of the microwells and channels had a width of 280 nm and a period of 535 nm. Figure 1d displays the tilted view and cross-section of the platform, demonstrating that the height of the nanoholes is 300 nm.

### 2.2. Fibronectin (FN) Coating

To coat the FN protein (Sigma-Aldrich, St. Louis, MO, USA) onto the PDMS platforms containing microwells and connecting channels with a flat bottom, the platforms were firstly hydrophilized by an O_2_ plasma, with conditions of 20 sccm, 250 mTorr, and 100 W rf power for 1 min. Then, the platforms were immersed in the FN solution, with a concentration of 50 μg/mL for 3 h at 4 °C. The excess FN was then rinsed with DI water and the FN-coated platforms were kept in phosphate buffered saline (1×, PBS, Gibco, Waltham, MA, USA) before cell seeding. Since the FN used in the experiments contained covalently linked rhodamine fluorescent dye, the uniformity of the FN coating could be checked using fluorescence images of the platforms. As depicted in Supplementary Figure S2, the FN was uniformly coated across all surfaces of the platforms. The brighter signals observed at the sidewalls was due to the accumulation of the FN fluorescence signals in the vertical direction.

### 2.3. Cell Culture

An Epstein–Barr virus (EBV)-positive NPC cell line (NPC43) was utilized in this project [32]. NPC43 cells were cultured in Roswell Park Memorial Institute medium 1640 (Gibco, Waltham, MA, USA), with 0.2% 2 mM rock inhibitor Y-27632 (ENZO, Farmingdale, NY, USA), 10% fetal bovine serum (FBS, Gibco, Waltham, MA, USA), and 1% antibiotic antimycotic (Gibco, Waltham, MA, USA; 100 units/mL penicillin G sodium, 100 mg/mL of streptomycin, and 0.25 mg/mL of amphotericin B). The NPC43 cells were maintained in an incubator at 37 °C, with a 5% CO_2_ supply. The medium was replaced every two days and the cells were passaged upon reaching 70% confluency. 

### 2.4. Time-Lapse Imaging and Treatment of the TGF-β Inhibitor

To prepare for cell seeding, the microwell and connecting channel PDMS platforms were mounted onto a plasma-treated confocal dish (SPL, Pocheon, Republic of Korea), followed by washing with 70% ethanol for sterilization. The platforms were then subjected to an O_2_ plasma treatment to enhance their hydrophilicity. The O_2_ plasma conditions were set to 20 sccm, 250 mTorr, and 100 W rf power for 1 min. To maintain the hydrophilicity of the platforms prior to cell seeding, PBS was added to the dish. For each experiment, a total of 1.5 × 10^5^ NPC43 cells in a 2 mL cell culture medium were evenly seeded onto the platforms. The PDMS platforms were fully submerged in the medium. The cells were loaded into the microwells with an open top to ensure a continuous supply of nutrients from the medium above the microwells. Before the time-lapse imaging, the NPC43 cells were incubated for 6 h to perform an initial attachment to the platforms and then the cell culture medium was changed to the mixture involving 1 mL NPC43 cell culture medium and 1 mL CO_2_ independent medium (Gibco, Waltham, MA, USA). The CO_2_ independent medium was supplemented with 1% GlutaMAX (100×, Gibco, Waltham, MA, USA), 10% FBS (Gibco, Waltham, MA, USA), and 1% antibiotic antimycotic (Gibco, Waltham, MA, USA; containing 100 units/mL penicillin G sodium, 100 μg/mL of streptomycin, and 0.25 μg/mL of amphotericin B). For the cell experiments with the TGF-β inhibitor, the galunisertib (Beyotime, Shanghai, China) TGF-β inhibitor was added into the medium, with a concentration of 10 μM, before time-lapse imaging. The time-lapse images were captured using an upright microscope (Eclipse NI-U, Nikon, Tokyo, Japan), at 5 min intervals, over a period of 16 h. The microscope was connected to a cell culture incubator to maintain the humidity level inside the imaging chamber to prevent the medium from evaporating.

### 2.5. Scanning Electron Microscopy

The NPC43 cells were fixed with 4% paraformaldehyde for 15 min, after seeding on the platforms for 22 h. The cells were immersed in PBS for 5 min, DI water for 10 min, and dehydrated using a series of ethanol solutions with increasing concentrations of 30%, 50%, 70%, 90%, 95% and 100%, with each step lasting for 5 min. Subsequently, the cells were dried using a critical point dryer (EM CPD300, Leica, Wetzlar, Germany), with a CO_2_ transitional medium. Before taking the images of the fixed NPC43 cells using a scanning electron microscope (SU5000, Hitachi, Tokyo, Japan), a thin layer of Au was coated onto the samples using a thin film coater (Q150 coater, Quorum Technologies, East Sussex, UK).

### 2.6. Cell Staining and Immunofluorescence Imaging

After seeding on the platforms for 22 h, the NPC43 cells were first fixed with 4% paraformaldehyde for 15 min. To permeabilize the cells, a 0.1% Triton X-100 solution (Thermo Fisher, Waltham, MA, USA) was applied for 10 min, followed by blocking the cells with 1% bovine serum albumin (Thermo Fisher, Waltham, MA, USA) for 30 min. The cells were then stained using an FAK100 actin cytoskeleton/focal adhesion staining kit (Merck, Burlington, MA, USA), using standard operating procedures, and maintained in PBS. The immunofluorescence images of the NPC43 cells were taken by a confocal microscope (STELLARIS 8, Leica, Wetzlar, Germany).

### 2.7. Data Analysis

The traversing probability of the NPC43 cells through the channels was defined as the percentage of microwells with channel units that had NPC43 cells migrated from one microwell through the channels to the opposite microwell. The cell migration trajectories and speed were analyzed by using the manual tracking function in ImageJ software (version 1.48). The cell travelling radius was defined as the distance from the cell seeding location to the furthest location the cell traveled during the 16 h time-lapse imaging. The cells that died or divided into multiple cells during the time-lapse imaging period were excluded from the analysis. The cell aspect ratio, solidity, and F-actin fraction were analyzed by using the measurement functions in ImageJ software. The aspect ratio is defined as the ratio between the major and minor axes. Solidity is defined as the total cell area divided by the cell convex area. The F-actin fraction is defined as the area of F-actin divided by the area of the cell outline. The cell protrusion length is defined as the distance between the cell edge and the end point of the protrusion. “N” represents the number of cells analyzed. Statistical analysis was performed using one-way analysis of variance (ANOVA) and Tukey’s post hoc test to determine the significance of the differences between the groups. The data were collected from at least three runs, and the results are presented as the mean ± standard error of the mean.

## 3. Results

### 3.1. Effect of the Channel Width on NPC43 Traversing Behaviors

#### 3.1.1. Reduced Traversing Probability with Smaller Channel Width

**Figure 2:**
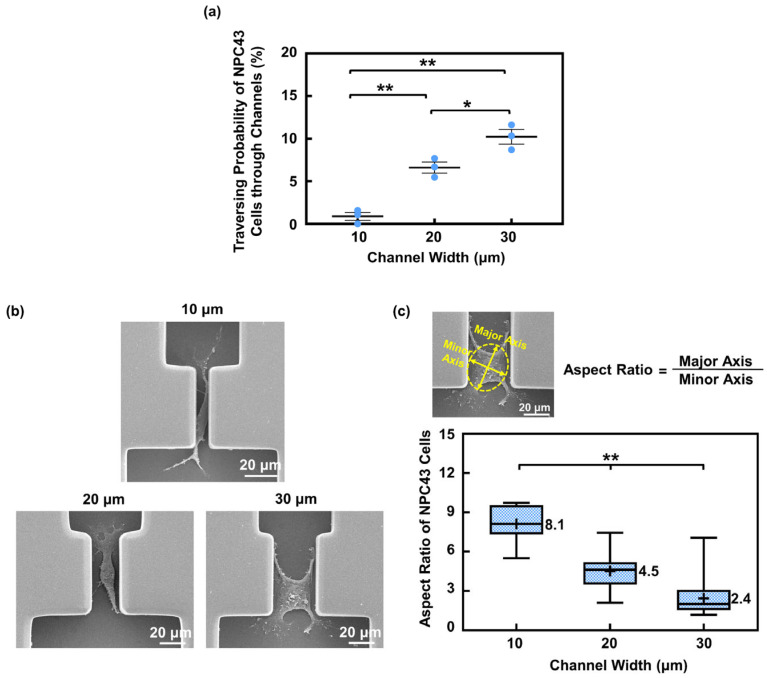
The channel width affected the traversing probability of NPC43 cells through the channels. (**a**) The traversing probability of NPC43 cells increased with larger channel widths. The surface consisted of flat bottoms. Each blue dot represents result from one round of experiment. (**b**) Micrographs of NPC43 cells in channels with a width of 10, 20, and 30 μm. (**c**) NPC43 cell outlined with best-fitted ellipse. Aspect ratio is defined as the ratio between the major and minor axes. The aspect ratio of NPC43 cells decreased with larger channel widths. Note: “+” represents mean value and “−” inside box represents median. One-way ANOVA and Tukey’s post hoc test with ** *p* < 0.01 and * *p* < 0.05.

The traversing behavior of the NPC43 cells in the microwells and connecting channels, with varying channel widths, was studied. The channel widths were designed to be 10, 20, and 30 μm to mimic the blood vessel and the confined microenvironment of the ECM. The platforms utilized in this section had a flat bottom without a coating and the NPC43 cells were left untreated, serving as typical platforms for the comparisons in the following sections. Due to the statistical variation in how cells randomly occupied the microwells, the cell number in different microwells varied. Typically, one to four cells were seeded in the 50 × 50 μm^2^ microwells and four to ten cells in 100 × 100 μm^2^ microwells. The results shown involved at least three rounds of experiments and the analysis of from 320 to 360 microwells with channels, with 1600 to 1900 cells monitored for their traversing probability through the channels. As depicted in Figure 2a, the traversing probability of the NPC43 cells decreased with the narrower channel widths. Specifically, the traversing probability of the NPC43 cells in 10, 20, and 30 μm wide channels were 0.9%, 6.6%, and 10.2%, respectively.

To successfully traverse through the connecting channels, the NPC43 cells departed from their seeding positions in the microwells to reach the channel region as the first step, and then deformed their cell bodies to migrate through the confined channels as the second step, as shown in Supplementary Video S1. The variation in the channel widths primarily influenced the number of NPC43 cells that could complete the second step, where the deformation ability of the cell bodies played a crucial role. The scanning electron micrographs (SEMs) of the NPC43 cells in the channels with different widths are shown in Figure 2b. The level of cell morphology deformation was quantified by the cell aspect ratio, which is defined as the ratio between the major and minor axes. As shown in Figure 2c, the aspect ratio of the NPC43 cells in the 10, 20, and 30 μm channels was 8.1, 4.5, and 2.4, respectively. The NPC43 cells experienced greater deformation in narrower channels, which led to a lower traversing probability in channels with narrower widths. 

#### 3.1.2. NPC43 Cell Nucleus Deformation inside Narrow Channels

**Figure 3:**
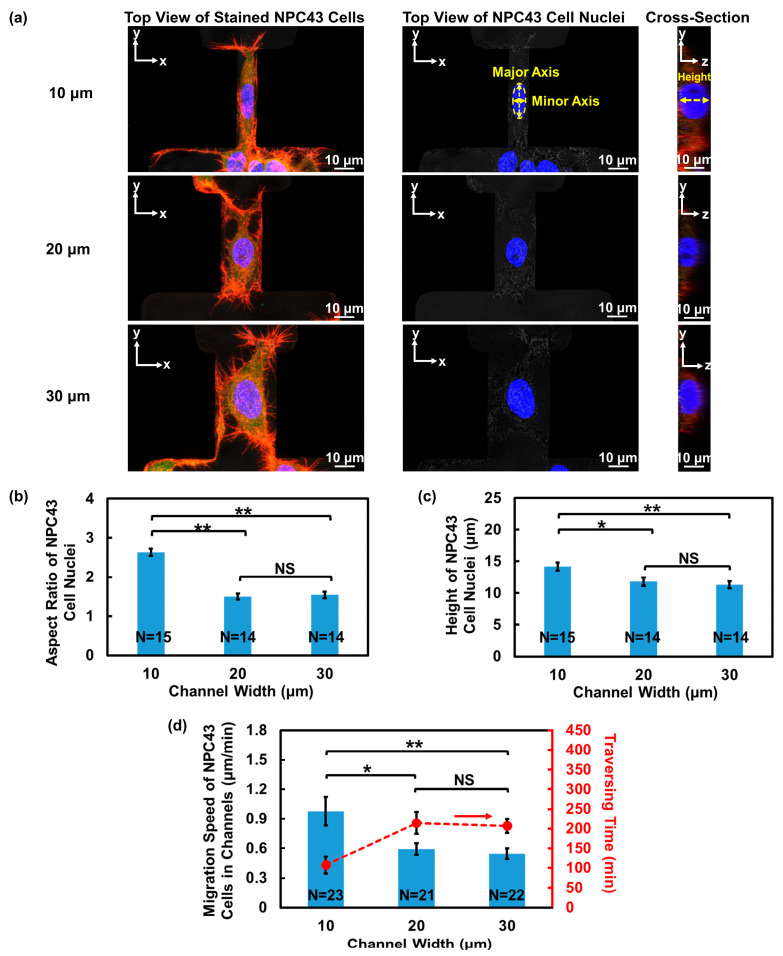
Nucleus shape of NPC43 cells in channels with different widths and microwells with flat bottoms. (**a**) Top view and cross-sectional view of fluorescence images of NPC43 cells in channels with different widths. NPC43 cells were stained to observe F-actin (red), nucleus (blue), and vinculin (green). Aspect ratio of nucleus was defined as the ratio between the major and minor axes. The height of nucleus was obtained from the cross-sectional image. (**b**) The NPC43 cell nucleus had the highest aspect ratio in 10 μm wide channels. (**c**) The NPC43 cell nucleus had the largest height in 10 μm wide channels. (**d**) The NPC43 cells had the highest migration speed (blue bars) and shortest traversing time (red dots) when migrating through 10 μm wide channels. One-way ANOVA and Tukey’s post hoc test with ** *p* < 0.01, * *p* < 0.05, and NS—not significant with *p* > 0.05.

The cell nucleus, being the largest organelle in the cell body, could affect cell behavior during migration through confined regions [33]. To investigate how the NPC43 cell nucleus responds to the confined microenvironment, 3D fluorescence imaging of the fixed cells was conducted, as depicted in Figure 3a. The top view of the NPC43 cell nucleus was analyzed to determine its aspect ratio, while the cross-sectional view was used to assess its height. Figure 3b,c illustrates that there was no significant difference in the cell nucleus aspect ratio and height between the 20 and 30 μm wide channels. However, in the 10 μm wide channels, the NPC43 cell nucleus exhibited a larger aspect ratio and height, suggesting that the NPC43 cell nucleus experienced suppression in the horizontal plane and became more elongated, along the vertical direction. Similar nucleus deformation was also observed during cancer cell invasion in vivo [34,35]. This additional deformation of the nucleus made it more difficult for the NPC43 cells to fit into the 10 μm channels, contributing to the lowest traversing probability. 

Despite having the lowest probability of passing through the 10 μm wide channels, the NPC43 cells exhibited the fastest movement within this highly restricted space, reaching the highest speed of 0.97 μm/min among all the channel widths, as shown in Figure 3d. There was no significant difference in the NPC43 cell migration speed when they moved through the 20 or 30 μm wide channels. Furthermore, the NPC43 cells had the shortest traversing time when passing through the 10 μm wide channels. This enhanced movement of the NPC43 cells in the 10 μm wide channels is likely due to the increased contact area between the cells and the channel sidewall surfaces, which facilitated NPC43 cell migration. In the 10 μm wide channels, the NPC43 cells not only interacted with the bottom surface, but also contacted the channel sidewall surfaces. In contrast, in the 20 or 30 μm wide channels, the NPC43 cells primarily spread across and migrated to the bottom surface. However, it is important to note that the influence of confinement on cell motility could change when the confinement is extremely tight [36,37]. Previous studies have mentioned that when the cross-sectional area of the confinement fell below a threshold and became very small, cell movement can be significantly suppressed and even stalled due to substantial nucleus deformation [38,39]. For instance, breast cancer cells showed decreased migration speed when the cross-sectional dimensions of the PDMS channels were reduced to 8 μm in width and 5 μm in height [38]. In the current study, the channels were designed with a height of 50 μm to effectively trap the cells. Although the NPC43 cell nuclei underwent deformation in the 10 μm wide channels, they did not reach the deformation threshold to impede the cell movement as the vertical restrictions on the cell nuclei were not significant.

### 3.2. FN Coating on Platforms Promoted NPC43 Cell Traversing Behavior

**Figure 4:**
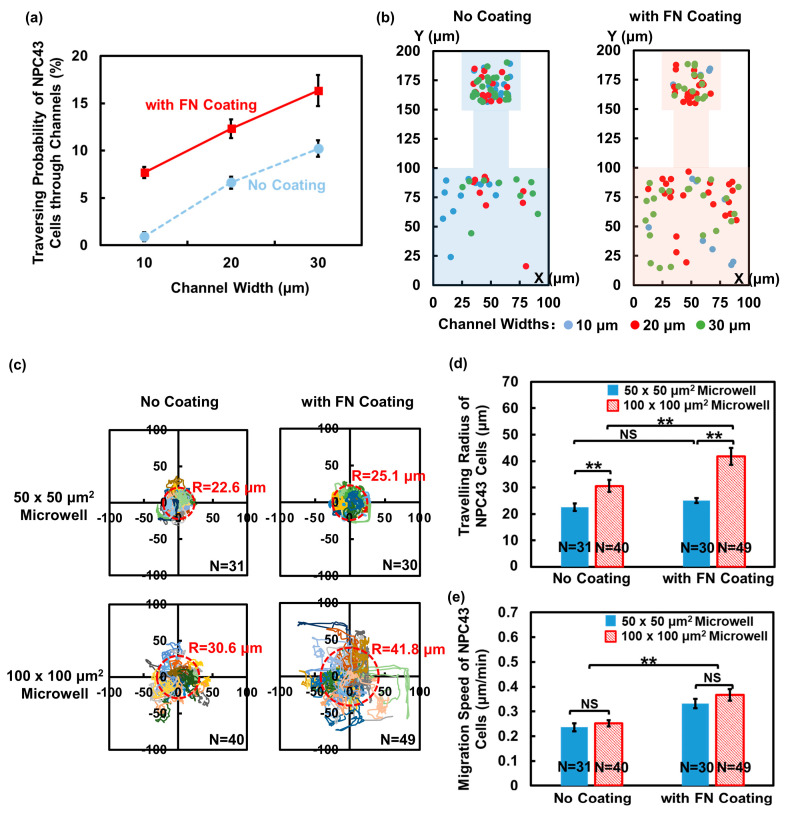
Impact of fibronectin (FN) coating on traversing behavior of NPC43 cells in flat-bottom microwells with connecting channels. (**a**) Traversing probability of NPC43 cells in platforms without and with FN coating. (**b**) Initial seeding positions of traversed NPC43 cells in platforms without FN coating and comparison to platforms with FN coating at t = 0 h. With FN coating, NPC43 cells located farther away from the channels were able to reach the channel region and finally traverse through the channels. (**c**) Migration trajectories of NPC43 cells in 50 × 50 and 100 × 100 μm^2^ microwells without and with FN coating. Trajectories of different colors represent migration of different cells. “R” represents travelling radius of NPC43 cells in microwells. (**d**) NPC43 cells had larger travelling radius in 100 × 100 μm^2^ microwells with FN coating. Smaller microwells limited the cell travelling radius. (**e**) NPC43 cells had higher migration speed in platforms with FN coating. Microwell size did not affect NPC43 cell migration speed. One-way ANOVA and Tukey’s post hoc test with ** *p* < 0.01 and NS—not significant with *p* > 0.05.

The behavior of cancer cells is influenced not only by the physical confinement of the ECM, but also the ECM proteins, such as FN. FN is a critical component of ECM structures. Through interactions with various cell surface receptors, FN plays a significant role in regulating cell adhesion and migration [40]. In order to investigate the impact of FN on the migration ability and traversing probability of NPC43 cells, FN was coated on the microwells and connecting channels. The introduction of the FN coating onto the platforms led to a significant increase in the traversing probability of the NPC43 cells compared to the ones without a coating. As illustrated in Figure 4a, the probability of the NPC43 cells traversing through channels of 10, 20, and 30 μm widths, with an FN coating, increased to 7.7%, 12.3%, and 16.3%, respectively, compared to 0.9%, 6.6%, and 10.2%, respectively, for platforms without an FN coating. 

#### 3.2.1. More NPC43 Cells Entered Microchannels Due to FN Coating

Figure 4b shows the initial seeding positions of the traversed NPC43 cells in the platforms without an FN coating and a comparison to the platforms with an FN coating. In the 50 × 50 μm^2^ microwells, cells in all positions had the opportunity to traverse through the channels, regardless of the presence of an FN coating. However, in the 100 × 100 μm^2^ microwells without an FN coating, most traversed cells were located within 50 μm from the channels. In contrast, in the 100 × 100 μm^2^ microwells with an FN coating, many of the NPC43 cells located farther than 50 μm away from the channels were able to reach the channels and eventually traversed through the channels. This increase in the distance between the initial seeding positions of the traversed cells and the channel openings indicated that the FN coating facilitated the first step of the NPC43 cells in traversing through the channels by assisting the cells in moving from their seeding positions to the channel openings. With the presence of the FN coating on the platforms, the NPC43 cells gained the ability to explore a larger area of the surrounding microenvironment, which attributed to the boosted cell migration behavior in response to the FN coating. To validate this, the migration trajectories and speed of the NPC43 cells in the microwells were analyzed. The investigation focused on analyzing the cell behaviors within the microwells, and the channel widths did not affect the analysis.

Figure 4c illustrates the migration trajectories of the NPC43 cells in the microwells without and with an FN coating. The travelling radius of the NPC43 cells in the microwells, denoted as “R”, represents the distance from the cell seeding location to the furthest location reached by the cell during the 16 h time-lapse imaging. As depicted in Figure 4c,d, the NPC43 cells consistently exhibited shorter migration trajectories and a smaller travelling radius in 50 × 50 μm^2^ microwells compared to 100 × 100 μm^2^ microwells, regardless of the presence of an FN coating. This observation can be attributed to the fact that NPC43 cells are more likely to come into contact with the sidewalls of 50 × 50 μm^2^ microwells, which acted as a barrier and limited the range of cell movement. In terms of the influence of the FN coating, the NPC43 cells showed extended migration trajectories and a significantly increased travelling radius in 100 × 100 μm^2^ microwells with an FN coating. However, there was no significant difference in the cell travelling radius in 50 × 50 μm^2^ microwells without and with an FN coating. These findings suggest that in loosely confined microenvironments where the cells had sufficient space to maneuver, the presence of an FN coating predominantly impacted the cell travelling range, while in more tightly confined microenvironments, the physical confinements played a decisive role. The combined effect of an FN coating and confinement determined the travelling range of the NPC43 cells. Figure 4e further demonstrates that NPC43 cells exhibited a higher migration speed in both 50 × 50 and 100 × 100 μm^2^ microwells with an FN coating, compared to those without an FN coating. Though the travel range of the NPC43 cells was restricted within the 50 × 50 μm^2^ confinement, the size of the microwells did not affect the cell migration speed. Unlike the narrow channel confinement, the level of confinement in the microwells did not induce notable changes in cell motility. The increased migration speed due to the FN coating facilitated the NPC43 cells in covering longer distances, thereby increasing the likelihood of entering narrow channels. 

#### 3.2.2. Enhanced Spread of NPC43 Cell Clusters Due to the FN Coating

**Figure 5:**
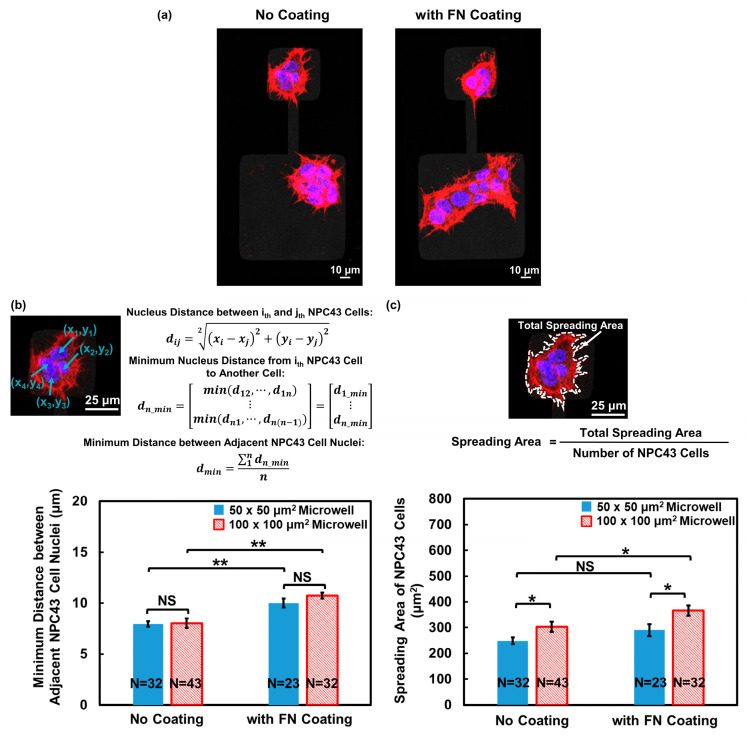
Effect of FN coating on NPC43 cell dispersion and spreading in microwells with flat bottoms. (**a**) Fluorescence images of NPC43 cell clusters on platforms without FN coating and comparison to platforms with FN coating. NPC43 cells were stained to observe F-actin (red) and nucleus (blue). (**b**) Minimum distance between adjacent NPC43 cell nuclei increased on platforms with FN coating. (**c**) Joint effect of FN coating and microwell size on spreading area of NPC43 cells. One-way ANOVA and Tukey’s post hoc test with ** *p* < 0.01, * *p* < 0.05, and NS—not significant with *p* > 0.05.

In addition to studying the cell migration behavior, the effect of an FN coating on NPC43 cell clustering was examined using fluorescence imaging, as shown in Figure 5a. Figure 5b demonstrates that the minimum distance between adjacent nuclei within the NPC43 cluster significantly increased in the microwells with an FN coating, indicating a more loosely packed cell–cell junction between the NPC43 cells in response to the FN coating. Furthermore, the NPC43 cells exhibited a larger spreading area in both 50 × 50 and 100 × 100 μm^2^ microwells with an FN coating compared to those without an FN coating, as shown in Figure 5c. The loosely packed arrangement of the NPC43 cells and their expanded spreading area in response to the FN coating also allowed them to explore a larger area of the surrounding microenvironment, which contributed to a higher probability of reaching the channels and eventually traversing through the channels.

#### 3.2.3. Comparison of Cell Motility in Channels without and with FN Coating

**Figure 6:**
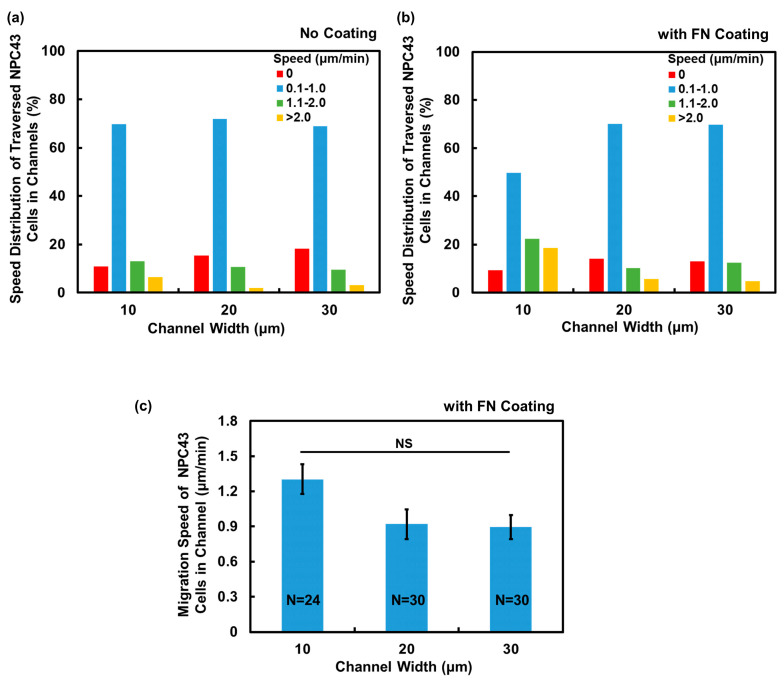
Comparison of NPC43 cell migration characteristics in channels without FN coating and comparison to channels with FN coating. Speed distribution of traversed NPC43 cells in (**a**) channels without FN coating and (**b**) comparison to channels with FN coating. Time-lapse images were captured over a total duration of 16 h. (**c**) NPC43 cells had higher migration speed in channels with an FN coating. One-way ANOVA and Tukey’s post hoc test with NS—not significant with *p* > 0.05.

After observing the enhanced migration ability of NPC43 cells in the microwells with an FN coating, the subsequent investigation focused on determining the potential impact of an FN coating on NPC43 cell migration through channels. The migration speed of the NPC43 cells through the channels, obtained every 5 min over 16 h, were calculated for the platforms without and with an FN coating, and the cell migration speed distributions are plotted in Figure 6a,b. The migration speed of the NPC43 cells through the channels, measured every 5 min, were categorized into four groups. Comparing Figure 6a,b, it is evident that the presence of an FN coating resulted in a higher percentage of migration speeds falling within the range > 2 μm/min across all channel widths, indicating an increase in the motility of NPC43 cells through the channels with an FN coating. The average migration speed of NPC43 cells through channels with an FN coating was further analyzed, as shown in Figure 6c. The results demonstrated that the average migration speed of the NPC43 cells increased to 1.30, 0.92, and 0.90 μm/min, respectively, with an FN coating and channels with 10, 20, and 30 μm widths, as compared to the channels without an FN coating, which exhibited speeds of 0.97, 0.59, and 0.55 μm/min, respectively. However, in regard to the 10 μm wide channels, no significant differences were observed. The NPC43 cell migration speed increased by 34% following an FN coating of the 10 μm wide channels. For the wider channels of 20 and 30 μm, the overall cell migration speeds were lower, but the percentage of the speed increase due to the FN coating was higher. These results suggest that the FN coating exerted a dominant effect on cell motility in a loosely confined microenvironment, while in a highly restricted area, the influence of physical confinement could partially override the impact of the FN coating.

### 3.3. Nanohole Topography Led to Reduced Cell Motility and F-Actin Formation

Extensive research has highlighted the significant influence of micrometer-sized topography cues generated by the ECM on cell behaviors. However, the impact of nanometer-sized structures is not clear and their effects on NPC cells remain unexplored. The basement membrane of the ECM is characterized by nanometer-sized features, such as holes, ridges, and fibers [41,42]. The hole structures were found to cover approximately 15% of the total area of the membrane surface [41]. Thus, nanoholes were distributed in a pattern on the bottom of the microwells and connecting channels to examine their influence on NPC43 cell behaviors.

**Figure 7:**
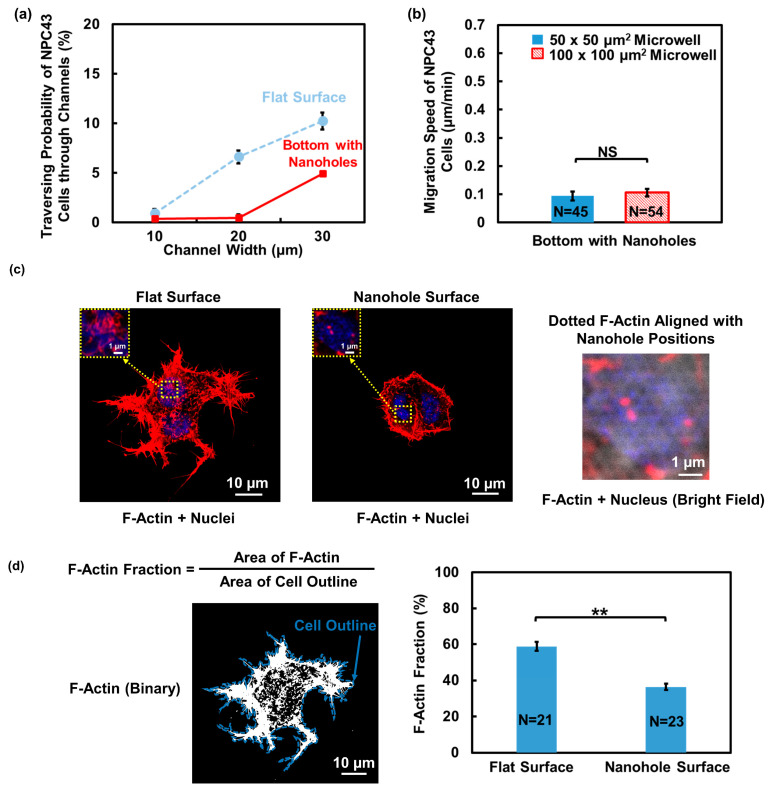
Effect of nanohole topography on the bottom of microwells with connecting channels on the traversing behavior and F-actin distribution of NPC43 cells. (**a**) Traversing probability of NPC43 cells in platforms with flat surface and with a pattern of nanoholes on the bottom. (**b**) NPC43 cells had lower migration speed in microwells with nanoholes compared to platforms with a flat bottom. Microwell size did not affect the migration speed. (**c**) F-actin in NPC43 cells on flat surface was denser, while F-actin on surface with nanoholes was sparse and formed some dotted structures. NPC43 cells were stained to observe the F-actin (red) and nucleus (blue). (**d**) F-actin fraction in NPC43 cells on surface with nanoholes was smaller. Fluorescence images of F-actin were converted to binary images, where the white areas represent the presence of F-actin structures. F-actin fraction is defined as the ratio between F-actin and the cell outline area. One-way ANOVA and Tukey’s post hoc test with ** *p* < 0.01 and NS—not significant with *p* > 0.05.

As shown in Figure 7a, the probability of NPC43 cells traversing through the channels with nanohole bottoms with a width of 10, 20, and 30 μm was 0.38%, 0.44%, and 4.93%, respectively. Compared to the platforms with a flat bottom, the traversing probability of the NPC43 cells decreased when the nanoholes were distributed in a pattern on the bottom. As the migration characteristics of NPC43 cells could affect the chance of the cells reaching the channel area, the migration speed of NPC43 cells in microwells with nanohole bottoms was examined, as shown in Figure 7b. The migration speed of NPC43 cells was 0.09 and 0.11 μm/min in 50 × 50 and 100 × 100 μm^2^ microwells with a pattern of nanoholes on the bottom. The microwell size did not affect the cell migration speed. Compared to the platforms with a flat bottom, where the migration speed of the NPC43 cells was 0.24 and 0.25 μm/min in 50 × 50 and 100 × 100 μm^2^ microwells, respectively, the presence of a nanohole topography pattern in the microwells led to a significant decrease in the cell migration speed. As shown in Supplementary Figure S3a, it is evident that the NPC43 cells exhibited shorter migration trajectories and a smaller travelling radius when migration took place in the microwells with nanoholes at the bottom. This restricted motility of NPC43 cells on the bottom with nanoholes hindered their ability to explore large areas or to reach the channel openings. Consequently, the traversing probability through the channels decreased due to these limitations in cell movement. 

Changes in the cell motility are often correlated with F-actin reorganization [43,44]. To investigate the cause of reduced motility of NPC43 cells on the nanohole topography, NPC43 cells cultured on the PDMS surfaces that were flat and had nanoholes were stained for immunofluorescence imaging to visualize the detailed organization of F-actin. A 63× oil lens for the confocal microscope was used to observe the nanoholes on the platform surface and achieve high-resolution images of F-actin structures. As shown in Figure 7c, the NPC43 cells exhibited denser F-actin distribution on the flat surface, whereas on the nanohole surface, the distribution was sparse and discontinuous. Notably, the nanohole surface induced some dotted F-actin structures that aligned with the position of the nanoholes and displayed a similar size as the nanoholes. This observation suggests that nanohole structures could trigger the rearrangement of F-actin in NPC43 cells. To quantify the F-actin changes on different surfaces, the F-actin fraction of NPC43 cells was analyzed. The F-actin fraction was defined as the ratio between the F-actin area and the cell area, serving as an indicator of the amount of F-actin present in the cells. Binary images were generated from the F-actin fluorescence images, as shown in Figure 7d, with white areas representing the presence of F-actin structures in NPC43 cells. A consistent thresholding method was applied to obtain binary images of the NPC43 cells on both the flat and nanohole surfaces. This method allowed for a comparative analysis of the F-actin fraction between the two surface conditions. The results demonstrated that the F-actin fraction in the NPC43 cells decreased from 58.8% on the flat surface to 36.4% on the nanohole surface, indicating the reduced generation of F-actin in the NPC43 cells on the nanohole surfaces. Previous studies have reported a positive correlation between F-actin content and cell migration speed [44,45]. Therefore, the reduced NPC43 cell migration speed on the nanohole surface may be attributed to the diminished formation of F-actin structures on the surfaces with nanoholes. These findings underscore the distinct impact of nanohole topography on both cellular F-actin formation and migration behaviors. Meanwhile, the spread of the NPC43 cells decreased on the nanohole surfaces, as shown in Supplementary Figure S3b. The reduction in cell spreading on the nanohole surface is consistent with the findings in previous reports [46,47].

### 3.4. Evaluation of TGF-β Inhibitor Downregulated NPC43 Cell Traversing Behavior

TGF-β inhibitor signaling plays an important role in tumor progression. Previous studies have shown that patients with NPC had a significantly higher level of active and total TGF-β cytokine in their serum compared to healthy individuals [48], indicating the involvement of TGF-β in NPC pathology. Galunisertib was identified as a selective TGF-β receptor I kinase inhibitor [49] and was investigated in both in vivo and in vitro models for its potential in treating various tumors, including hepatocellular carcinoma [50] and ovarian cancer [51]. However, it is still unclear whether the TGF-β inhibitor galunisertib has an effect on NPC cells. To investigate the potential therapeutic implications of targeting the TGF-β pathway in NPC in vitro, we utilized the platforms with a flat bottom to perform a detailed evaluation of the NPC43 cell migration behavior and metastatic potential after the addition of the TGF-β inhibitor. To focus on the administration of galunisertib and assess its specific impact on NPC43 cell behaviors, NPC43 cells were treated with galunisertib alone without adding exogenous TGF-β. Galunisertib was added into the cell culture medium at the beginning of the time-lapse imaging, and its influence on the traversing behavior of NPC43 cells in microwells and connecting channels was studied.

**Figure 8:**
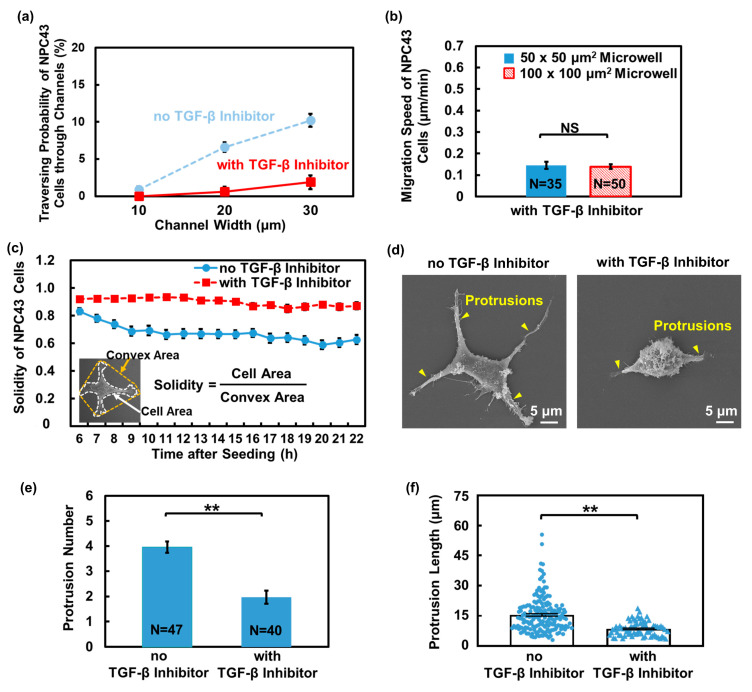
Effect of TGF-β inhibition on traversing behavior of NPC43 cells in microwells with a flat bottom. (**a**) Traversing probability of NPC43 cells without and with TGF-β inhibitor treatment in platforms with a flat bottom. (**b**) Migration speed of NPC43 cells decreased with the addition of TGF-β inhibitor. Microwell size did not affect the migration speed. (**c**) During 16 h time-lapse imaging, the solidity of NPC43 cells remained unchanged in response to TGF-β inhibitor, while without the TGF-β inhibitor, the solidity of the NPC43 cell decreased. Solidity is defined as the cell area divided by the convex area. (**d**) Micrographs of NPC43 cell without and with TGF-β inhibition. (**e**) Number of protrusions of NPC43 cells decreased with the TGF-β inhibitor addition. (**f**) Protrusion length of NPC43 cells decreased with the TGF-β inhibitor addition. Each blue dot or triangle represents length of one protrusion. One-way ANOVA and Tukey’s post hoc test with ** *p* < 0.01 and NS—not significant with *p* > 0.05.

Figure 8a demonstrates the traversing probability of NPC43 cells without and with the TGF-β inhibitor treatment in microwells and connecting channel platforms with a flat bottom. In comparison to the untreated cells, a marked decrease in the traversing probability of NPC43 cells was observed upon the addition of the TGF-β inhibitor. When treated with the TGF-β inhibitor, the migration speed of NPC43 cells also decreased. Specifically, in the 50 × 50 and 100 × 100 μm^2^ microwells, the migration speed of untreated NPC43 cells decreased from 0.24 and 0.25 μm/min to 0.15 and 0.14 μm/min, respectively, upon treatment with the TGF-β inhibitor, as shown in Figure 8b. Supplementary Figure S4a shows that treatment with the TGF-β inhibitor significantly reduced the range of the migration trajectories and the average travelling distance of the NPC43 cells in both the 50 × 50 and 100 × 100 μm^2^ microwells, compared to the untreated cells. Similar to the case of NPC43 cells on the nanohole surface, the decrease in the motility limited the range of movement of the NPC43 cells within the microwells, thereby reducing their probability of reaching the channels. To illustrate whether this decrease in NPC43 cell migration motility following treatment with the TGF-β inhibitor was also associated with changes in F-actin structures, immunofluorescence imaging was performed on the treated NPC43 cells cultured on the flat PDMS surface, as shown in Supplementary Figure S4b. It was found that the F-actin fraction in the NPC43 cells treated with the TGF-β inhibitor was smaller compared to those without treatment on a flat surface, decreasing from 58.8% to 50.4%. Although the reduction in the F-actin fraction of the NPC43 cells treated with the TGF-β inhibitor is smaller compared to those on the nanohole surface, it partially explains the decrease in cell motility observed with the TGF-β inhibitor treatment. These findings suggest that the dense network of F-actin plays a critical role in facilitating NPC43 cell motility. The disruption of the F-actin structure and decrease in the amount of F-actin may contribute to the reduced motility of NPC43 cells. 

It is well-established that the activity of cell protrusions is influenced by TGF-β, which in turn affects cell motility [52]. Apart from the F-actin changes, the protrusion formation of NPC43 cells with the TGF-β inhibitor treatment was also examined. To accurately quantify the changes in the protrusion formation induced by the TGF-β inhibitor, the NPC43 cells were seeded on the flat PDMS surface. Changes in the shape of the NPC43 cells were monitored over a 16 h time-lapse imaging period and the protrusion formation was quantified using cell solidity, which is defined as the cell area divided by the cell convex area. Generally, a decrease in solidity indicates the presence of more and longer protrusions extending from the cell body, indicating a deviation from the convex hull. Figure 8c shows that the solidity of NPC43 cells without treatment with the TGF-β inhibitor gradually decreased from 0.83 to 0.62 over time. Initially, the NPC43 cell had a more rounded shape with little protrusions, resulting in larger solidity values. Over time, the NPC43 cells formed more stable attachments and protrusions stretched out in all directions, leading to a decrease in solidity. In contrast, when treated with the TGF-β inhibitor, the NPC43 cells exhibited consistent solidity of approximately 0.9 throughout the time-lapse imaging period. The majority of cells formed fewer protrusions upon treatment with the TGF-β inhibitor. To quantify the changes in the number and length of protrusions in NPC43 cells without and with treatment with the TGF-β inhibitor, SEM images were obtained, as shown in Figure 8d. With the TGF-β inhibitor treatment, both the protrusion number and length of the protrusions in the NPC43 cells decreased, as shown in Figure 8e,f, further confirming that the ability of the NPC43 cells to form protrusions was weakened under the influence of the TGF-β inhibitor.

Overall, the impaired ability to form protrusions and the limited motility resulting from the treatment with the TGF-β inhibitor made it challenging for the NPC43 cells to reach the channel openings and traverse through the channels, leading to a substantial decrease in the traversing probability through the channels. These findings highlight the impact of TGF-β inhibitor signaling in regulating the shape and motility of NPC43 cells and suggest that the TGF-β inhibitor galunisertib could be a potential drug for NPC treatment. 

## 4. Discussion and Conclusions

The significant disparity between the highly intricate in vivo microenvironment of tumors and conventional cell culture dishes necessitates the development of a more physiologically relevant model to efficiently study cancer development and metastasis. Previous studies have utilized microwell platforms of various sizes and shapes to investigate cell migration and interactions in confined microenvironments [16,26,53]. In contrast to prior work, this study aims to modify the platforms by incorporating nanotopography and protein coating, thereby creating a more realistic model that mimics the chemical and physical complexities of the ECM. To the best of our knowledge, this is the first report showing nanotopography integrated into microwell platforms, demonstrating the importance of nanotopography on the migration behavior of NPC cells. The fabrication technology developed in this work holds great potential for widespread applications in 3D biosystems and microfluidics. It will pave the way for more comprehensive cell studies focusing on the effects of topography and surface chemistry, thereby promoting our understanding of cellular behaviors in complex microenvironments.

With the established microwells and connecting channel platforms, the metastatic potential of NPC43 cells was investigated by assessing the traversing probability of NPC43 cells through confined channels. The NPC43 cells showed lower traversing probability in channels with narrower widths, highlighting the effect of confinement on cell translocation. As it is more challenging for NPC43 cells to undergo larger deformations in narrower channels, with their traversing probability being reduced. This indicates that the deformation ability of cells plays an important role in the metastasis process. Further investigation into the mechanisms underlying cell deformation could be useful for developing control therapies for NPC metastasis. Additionally, the NPC43 cells demonstrated the fastest movement within the narrowest 10 μm wide channels, likely due to the increased contact area between the cells and the channel sidewalls. These findings emphasize the influence of a confined environment in simultaneously reducing the cell traversing probability, while promoting cell motility. Understanding how cells respond to confined spaces could provide valuable insights into the cancer metastasis cascade. By manipulating the cellular microenvironment, it will be possible to modulate cell behaviors and potentially inhibit the metastatic process.

Although the effect of FN on cell migration has been studied, the effect of FN on NPC43 cells in confined microenvironments remained unexplored. The results showed that an FN coating promoted the traversing probability of NPC43 cells by enhancing cell motility, dispersion, and spreading within the microwells, highlighting the crucial role of FN coatings in microenvironments in facilitating cell movement and expansion. Moreover, the influence of FN on NPC43 cell migration was not limited to their presence in microwells, but also extended to connecting channels. The NPC43 cells exhibited higher migration speeds inside the connecting channels compared to inside the microwells due to the greater channel confinement, which resulted in additional cell elongation that facilitated cell movement. The presence of FN further enhanced the migration speed of NPC43 cells in the channels, showing the combined effect of the FN coating and physical confinement in promoting cell motility. These findings suggest that FN plays an important role in NPC progression and could be a potential target for NPC treatment. 

On the other hand, the nanohole topography patterned on the bottom of the platform led to a decreased traversing probability of NPC43 cells through the channels. The NPC43 cells showed significantly reduced cell motility on the nanohole surface, which can be attributed to the diminished F-actin generated in the NPC43 cells on the nanohole surface. This decrease in F-actin levels hindered the cells’ ability to produce contractile forces necessary for cell elongation and contraction [54,55], thereby limiting efficient cell movement. Moreover, the reduction in F-actin also likely contributed to the decreased traversing probability of cells in 10 and 20 μm wide channels with nanoholes compared to 30 μm wide channels. This is because cells require greater cell deformation to pass through narrower channels. The reduced F-actin in the cells on the nanohole surface impaired their ability to undergo the necessary deformation to efficiently traverse the narrower 10 and 20 μm wide channels. Although nanopillar topography has been shown to affect cell behaviors [18,56], its effect on NPC43 cells was not as profound as that of nanoholes. Therefore, this study focused on the exploration of the impact of nanohole topography. The findings emphasize the regulatory role of nanohole topography on NPC43 cell behavior and could provide valuable insights for the development of next-generation biomaterials for medical and implanted devices, where surface modifications with nanoholes can be employed to control NPC cell behavior. The size and depth of the nanoholes could be further tailored to achieve the desired impact on cell migration. A previous study has demonstrated that osteoblastic cells exhibited a reduced migration speed and elongation when they were on a surface with nanoholes with a larger depth. This effect was attributed to more cell protrusions being trapped in the deeper nanoholes, leading to decreased cell motility [18]. The nanohole size was found to affect cell spreading, elongation, and proliferation [47,57]. These observations collectively demonstrate the ability of nanoscale topography in engineered biomaterials to modulate cell behaviors. By optimizing the nanotopography, including the depth, size, and density of nanostructures, it is possible to manipulate and guide cell migration behaviors.

Given the contrasting effects observed between FN coatings and nanohole topography on NPC43 cell traversing probability and motility, a valuable future direction is to elucidate how these two distinct biochemical and biophysical cues could jointly influence cellular behaviors when they are simultaneously present on such platforms. Unraveling these complex interactions between the cells and the combined effects of FN coatings and nanotopography will be instrumental in identifying the key determinants to control NPC cell migration, which is of importance for developing improved cancer therapeutic strategies.

It was reported that an elevated level of TGF-β was detected in the serum of patients with EBV-associated NPC, potentially originating from tumor cell secretion and EBV infection [48]. This finding indicated that TGF-β was involved in NPC progression. Currently, the TGF-β signaling pathway is recognized as a promising target for treating various cancers, prompting studies to explore its impact on NPC cell behaviors. The specific impact of TGF-β on NPC varied with the NPC cell line used. It was found that the growth of the NPC cell line CNE2 was unaffected by TGF-β1 levels [58]. Another study showed that the TGF-β1 inhibitor could partially inhibit NPC cell migration and invasion induced by IncRNA POU3F3 overexpression [59]. However, the effect of TGF-β inhibition on the NPC43 cell line remains unexplored. Therefore, the proposed platforms were employed to investigate the impact of the TGF-β inhibitor galunisertib on the migration and traversing behaviors of NPC43 cells. The experimental findings demonstrated a noteworthy reduction in the traversing probability, suggesting that galunisertib may have the potential to inhibit NPC43 metastasis. The inhibition of TGF-β not only resulted in a decreased F-actin fraction within NPC43 cells, but also impaired the ability of NPC43 cells to form long protrusions, leading to reduced cell motility. 

Moreover, these platforms hold promise for assessing the metastatic potential of other cancer cell types and evaluating the efficacy of potential drugs in suppressing metastasis. The NPC43 cell line used in this work was an EBV-positive cell line [32]. In clinical scenarios, EBV-positive NPC cases typically demonstrated a higher level of invasiveness and metastatic potential compared to those not linked with EBV [60]. Given the genetic heterogeneity across various NPC cell lines, it is plausible that the results could differ when extrapolated to cell lines with distinct genetic backgrounds. Previous research has indicated that NPC cell lines derived from different patients displayed varying degrees of motility and morphological heterogeneity [61]. Given the significant influence of the cell migration speed and morphology changes on the traversing probability of cells through channels, the likelihood of different cell lines traversing through the microchannels in the proposed platforms could vary accordingly. By seeding NPC cells with diverse genetic backgrounds into the platforms, it is possible to investigate how genetic variations could influence NPC cell metastatic behavior and responses to biophysical and biochemical stimuli, thereby offering a more comprehensive understanding of NPC characteristics.

In conclusion, the development of 3D microwells and channels platform in this work provides a promising approach for in vitro analysis of cancer cell metastasis behavior and drug screening. Diverse types of topographies and proteins can be easily integrated into these platforms using methods demonstrated in this work. Moreover, these results have enhanced our fundamental understanding of NPC metastasis and have provided insights for the development of therapies for NPC.

## Data Availability

The original contributions presented in the study are included in the article/Supplementary Materials, further inquiries can be directed to the corresponding author.

## Supplementary Materials

The following supporting information can be downloaded at: https://www.mdpi.com/article/10.3390/jfb15090263/s1, Figure S1: Fabricated PDMS platform containing microwells with connecting channels; Figure S2: Fluorescence images of FN-coated microwells with connecting channel widths varying from 10 to 30 μm. Fabricated PDMS platform containing microwells with connecting channels; Figure S3: NPC43 cells had shorter migration trajectories and a smaller traveling radius in microwells with nanoholes; Figure S4: NPC43 cells had shorter migration trajectories and a smaller travelling radius in 50 × 50 and 100 × 100 μm^2^ microwells with TGF-β inhibitor addition; Video S1: NPC43 cells traversed through 20 μm wide channel.
