# Supplementary material for: Effects of Confined Microenvironments with Protein Coating, Nanotopography, and TGF-β Inhibitor on Nasopharyngeal Carcinoma Cell Migration through Channels
Source: J Funct Biomater. 2024 Sep 11;15(9):263. doi: 10.3390/jfb15090263 (PMC11433299; doi:10.3390/jfb15090263)
Supplement: Supplementary file 1 [file jfb-15-00263-s001.zip › Confined Cell Migration-Supplementary Materials.pdf]

Electronic Supplementary Information (ESI)

Effects of Confined Microenvironments with Protein Coating, Nanotopography, and TGF-β Inhibitor on Nasopharyngeal Carcinoma Cell Migration through Channels

X. Hong, Y. H. Xu, and S. W. Pang*
Department of Electrical Engineering
Centre for Biosystems, Neuroscience, and Nanotechnology
City University of Hong Kong, Hong Kong, China

*Corresponding Author:
S. W. Pang (pang@cityu.edu.hk)
Department of Electrical Engineering
City University of Hong Kong
83 Tat Chee Avenue, Kowloon
Hong Kong, China
Phone: +852 3442 9853
Fax: +852 3442 0562

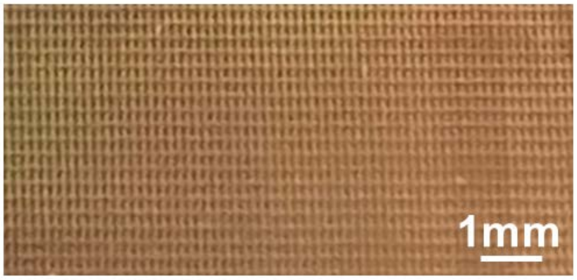

Supplementary Figure S1. Fabricated PDMS platform containing microwells with connecting channels.

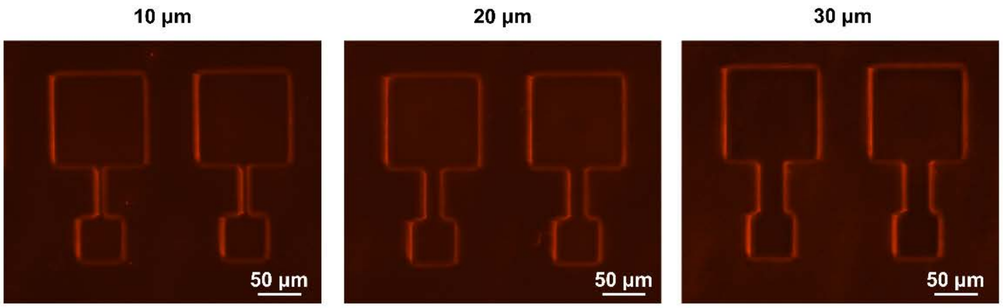

Supplementary Figure S2. Fluorescence images of FN-coated microwells with connecting channel widths varying from 10 to 30 μm. FN contained covalently linked rhodamine fluorescent dye and it was uniformly coated on platforms.

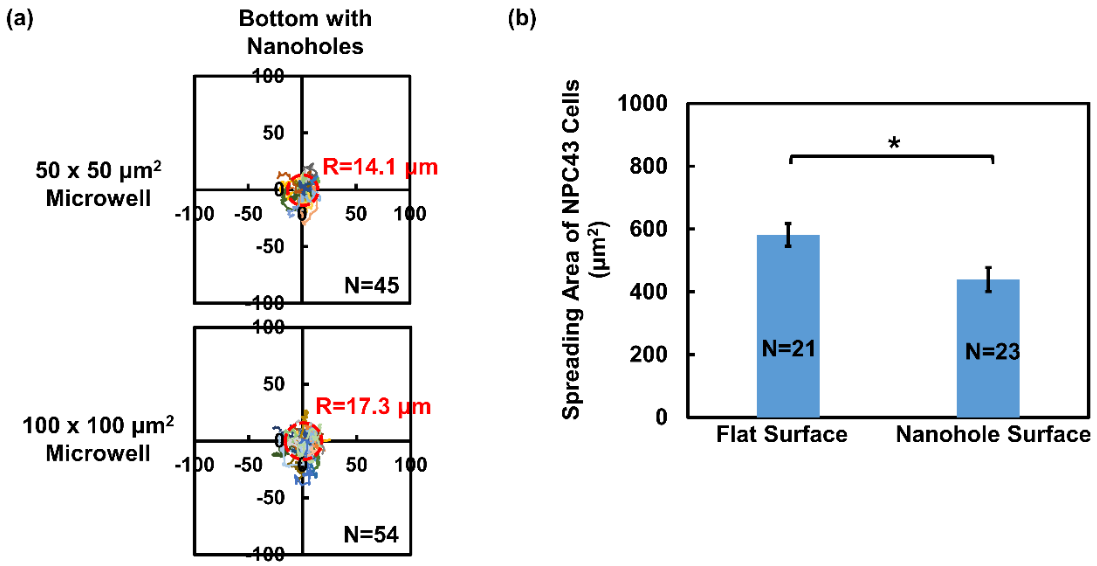

Supplementary Figure S3. (a) NPC43 cells had shorter migration trajectories and smaller travelling radius in microwells with nanoholes. “R” represents travelling radius of NPC43 cells in microwells. (b) Spreading area of NPC43 cells decreased on nanohole surface. One-way ANOVA and Tukey's *post hoc* test with *p <0.05.

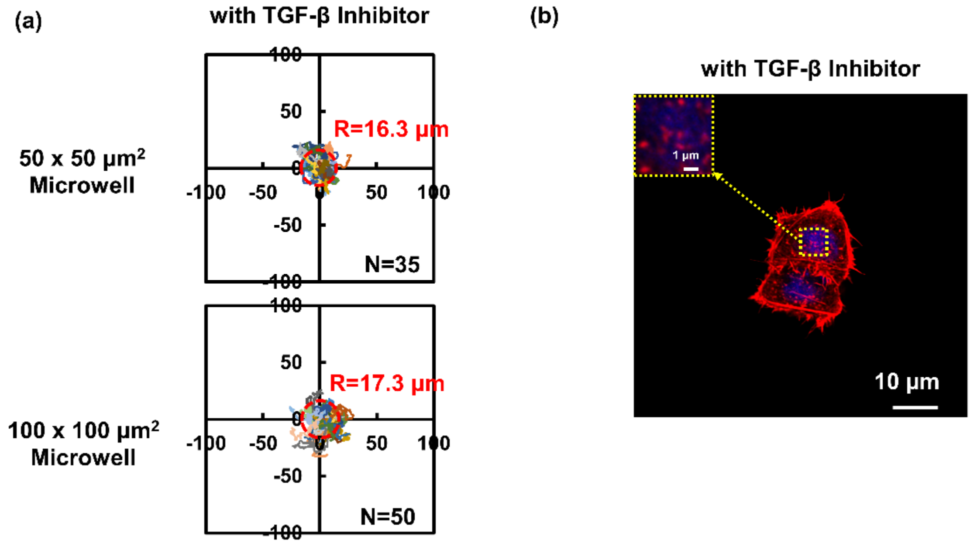

Supplementary Figure S4. (a) NPC43 cells had shorter migration trajectories and smaller travelling radius in 50 x 50 and 100 x 100 μm^2 microwells with TGF-β inhibitor addition. "R" represents travelling radius of NPC43 cells in microwells. (b) F-Actin of NPC43 cells treated with TGF-β inhibitor. Fluorescence image contains two cells. NPC43 cells were stained to observe the F-Actin (red) and nucleus (blue).
